# Asymmetric strategy for enhanced performance of flexible electroadhesive clutch

**DOI:** 10.1016/j.heliyon.2023.e12938

**Published:** 2023-01-12

**Authors:** Jun Li, Ying Xiong, Kitming Ma, Bao Yang, Linlin Ma, Xiaoming Tao

**Affiliations:** Research Institute for Intelligent Wearable Systems, School of Fashion and Textiles, The Hong Kong Polytechnic University, Hung Hom, Hong Kong, China

**Keywords:** Electroadhesive clutch, Fabric, Polylactic acid, PDFE, Asymmetric

## Abstract

Flexible electroadhesive clutches with high shear stress and fast response working at low voltage are much desired in wearable electronics and robotic systems. Dielectric materials with opposite charge characteristics could maximize the clutch performance by taking advantages of the boosted electroadhesion between the two contact pads in an asymmetrically structured clutch. In this paper, asymmetrically structured electroadhesive clutches are proposed and reported for the first time. The asymmetric structured clutch exhibits a two-fold increment in the shear force but similar response time by simply reversing the electrode polarity. This work provides a new dimension to realize high-performance electroadhesive clutches based on an asymmetric strategy.

## 1 Introduction

Clutches play important roles in robotics [1], actuators [[2], [3], [4]] and haptics applications [5,6] by mechanical actions. Traditional clutches, made of electromagnetic solenoids [7], magnetorheological fluids [8,9] and electrorheological materials [10] are bulky and heavy with high power consumptions up to several hundred watts. Working with a mechanism of electroadhesion between dielectric materials, a new family of electroadhesive clutches has emerged in recent years. Such a clutch normally comprises two electroadhesive pads, each equipped with a dielectric layer and a planar electrode. The two pads adhere to each other when a DC voltage is applied, and release after the removal of the field. Electroadhesive clutches have found promising applications in wearable devices [11–13] and haptic feedback systems [14,15] where dynamic blocking is needed, due to their high compatibility with electrical systems, fast responsiveness, energy-saving and lightweight.

The previously reported electroadhesive clutches all have a symmetric structure, that is identical dielectric materials are used with the positive and negative electrodes. Using high dielectric constant (high-*k*) polymers [15] or composites [12] as the functional layers has been demonstrated to be effective to increase the adhesive force at lower working voltage. However, high-*k* composites may draw safety concern due to the arc discharge or sparks caused by the low breakdown strength [11], while the high-*k* polymers are expensive with very limited species. There is an increasing demand for developing high performance electroadhesive clutch with wide adaptability, diverse material selection and safety to satisfy requirements for different application scenarios.

The charge characteristics of dielectric materials should play important roles in the electroadhesion. Considering the electrostatic attraction between two surfaces of difference electron affinity, opposite charges are induced under an external electric field. An alternative way to enhance the electroadhesion is to explore the asymmetric structure using dielectric materials with opposite electron affinity. According to our previous findings, bio-polyesters, such as polylactic acid (PLA), have a high positive charge density and are likely to lose electrons, while many other synthetic polymers like polyimide (PI) tend to gain and keep electrons [16].

In this paper, we propose a novel asymmetrically structured flexible electroadhesive clutch made from polymers with opposite electron affinity, i.e., PLA and PI. The electroadhesion performance of asymmetric structured clutches are evaluated and compared with those of symmetric ones. A direct method is adopted for the measurement of response time of the electroadhesive clutches. Apart from using high-*k* materials, this work illustrates an alternative approach to improve the clutch performance and the asymmetric strategy could enable a more diverse materials selection for the clutch design.

## 2 Results and discussion

### 2.1 Clutch structure

**Fig. 1:**
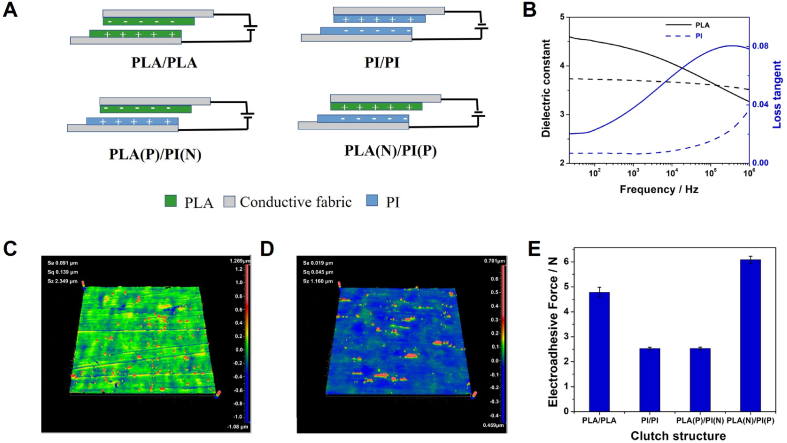
Clutch structures and material characterization. (A) Schematic illustration of the device structure of the symmetric and asymmetric electroadhesive clutches; (B) Frequency dependent relative dielectric constant and loss tangent of PLA and PI; Surface roughness of (C) PLA and (D) PI film; Electroadhesive forces of clutches with different structures (V = 600 V, A = 4 cm^2^ and t = 50 μm).

The schematic illustration of the symmetric and asymmetric clutches is shown in Fig. 1A. Unlike symmetric clutches with two identical electroadhesive active pads, in the asymmetric clutches, two active pads are made from dielectric materials with different electron affinity. The PI and PLA films are directly adhered on the electrically conductive fabrics.

### 2.2 Dielectric properties of the active pad materials

Fig. 1B shows the dielectric properties of PLA and PI. PLA exhibits a higher dielectric constant and loss than PLA due to the increased molecular polarity. PLA has the top high positive surface charge density in the triboelectric series of most polymers. When the frequency increases, the dielectric constant of PLA decreases slightly and PI almost remain constant. PI exhibits a lower dielectric loss as compared with PLA due to its relatively low molecular polarity [17,18]. The principal polarization should be dipolar polarization for both PI and PLA [19].

### 2.3 Surface morphology of the active pads

The surface morphologies and roughness of the PI and PLA films were evaluated by the SEM (see Figure S1) and surface roughness (see Fig. 1C and D), where Sa, Sq and Sz are arithmetical mean, root mean square and maximum height of the films. These results show that the average surface roughness of PLA and PI are 91 nm and 19 nm respectively. The PLA and PI films show very smooth surfaces as shown in the SEM images. The frictional coefficients of the PLA and PI films were 0.43 and 0.38.

### 2.4 Symmetric clutches made from PLA and PI

Symmetric electroadhesive clutches made from PLA and PI have been prepared for comparison and the generated electroadhesive forces have been evaluated (see Fig. 1E). The working voltage was fixed at 600 V. The thickness of each dielectric layer and overlapping area was set as 25 μm and 4 cm^2^. The PLA/PLA clutch could generate an electroadhesive force of 4.78 ± 0.21 N while PI/PI could provide a shear force of 2.53 ± 0.05 N. The enhanced shear force of the PLA/PLA clutch should be ascribed to the relatively high friction ecoefficiency and dielectric constant of PLA layer.

#### 2.4.1 Response time

Current method for measuring the clutch response time generally based on capacitor charging/discharging [11], which is an indirect method and only the electric factors are considered. However, other factors that may influence the response time of the clutches such as vacuum adhesion and interfacial interactions could not be evaluated. In this part, a direct method was proposed. The circuit design for the experimental setup was demonstrated in Figure S2. The engage time is described as the timeslot between the turn-on of external power and generation of the electroadhesive force. And the release time refers to the duration of the power turn-off to 10% of the maximum holding force. The electroadhesive forces, engage and release time of the four sets of clutches are summarized in Table 1.

**Table 1:** Clutch performance of symmetric and asymmetric clutches made from PLA and PI (E = 600 V, t = 50 μm, A = 4 cm^2^).

Clutch structure	Electro-adhesive force/N	Direct method		Indirect method		Current/nA
		Engage time/ms	Release time/ms	Engage time/ms	Release time/ms	
**PLA/PLA**	4.78 ± 0.21	18.25 ± 2.06	43.27 ± 4.90	8.4 ± 0.55	8.8 ± 0.45	123.12 ± 2.22
**PI/PI**	2.53 ± 0.05	47.50 ± 2.51	73.70 ± 3.49	8.2 ± 0.44	8.8 ± 0.44	104.49 ± 2.82
**PLA(P)/PI(N)**	2.54 ± 0.05	23.32 ± 2.52	43.50 ± 3.44	8.8 ± 0.84	8.8 ± 0.84	102.26 ± 0.78
**PLA(N)/PI(P)**	6.09 ± 0.14	15.50 ± 1.29	43.47 ± 1.75	9.8 ± 0.84	10.0 ± 0.71	97.04 ± 0.79

**Fig. 2:**
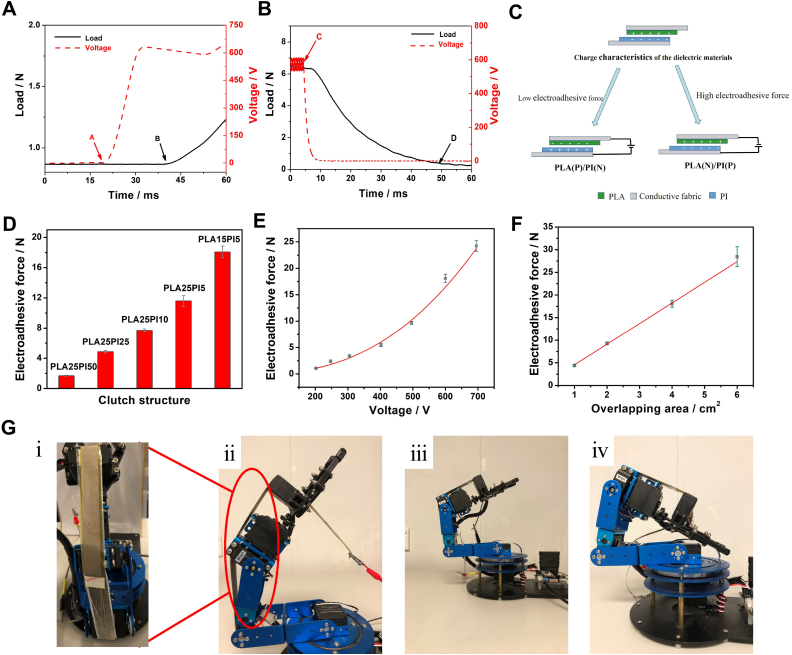
Performance and mechanism of the symmetric and asymmetric clutches. (A) Typical engage and (B) release time of the asymmetric clutch; (C) two connection methods in asymmetric-structured clutches; (D) electroadhesive force of PLA(N)/PI(N) clutch with various dielectric thicknesses (V = 600 V and A = 4 cm^2^); (E) electroadhesive force of PLA15PI5 clutch vs. voltage (A = 4 cm^2^); (F) electroadhesive force of PLA15PI5 clutch with various overlapping areas (V = 600 V); (G) demonstration of the electroadhesive clutch on a robotic arm. (i) Enlarged picture of the clutch; (ii) large holding angle at 600 V; (iii) small holding angle at 600 V; (iv) V = 0 V.

The typical pictures for engage and release time of the PLA(N)/PI(P) clutch are shown in Fig. 2A and B. Point A is the switch of the power source and at point B electroadhesion initials. In Fig. 2B and C is the switch-off point of the applied voltage and the holding force drop to 10% of the maximum value at point D. The release time of the asymmetric clutches is about 40 ms which is higher than the literatures (∼20 ms) [11,12]. It should be noticed that the release time may be limited by the dynamic of the load cell in this circumstance. The indirect method based on capacitor charging/discharging [11] have also been conducted for comparison (see Figure S3) and all clutches exhibit response time below 10 ms. The data are summarized in Table 1.

### 2.5 Factors influencing performance of the asymmetric PLA/PI clutches

In this section, the influencing factors of its electroadhesive force were experimentally investigated, including applied external voltage, dielectric thickness, overlapping area. Asymmetrical structured PLA(N)/PI(P) and PLA(P)/PI(N) clutches have been developed and their electradhesive forces were determined. PLA(N)/PI(P) generated an electroadhesive force of 6.09 ± 0.14 N at 600 V with an overlapping area of 4 cm^2^, which is higher than those from PLA/PLA, PI/PI and PLA(P)/PI(N) clutches under same conditions, indicating the advantages of the asymmetric strategy. When the PLA pad was connected to negative pole, the asymmetric clutch could generate a higher electroadhesive force as compared with the structure with PLA at positive side. The PLA(N)/PI(P) clutch could generate a shear stress of 15.22 ± 0.35 kPa at 600 V. This result indicates that the inherent charge affinity of the dielectrics is conducive to the shear force as well in asymmetrical structures. It will result in an increased shear force if the induced charge polarity is consistent with charge affinity of dielectric layers (see Fig. 2C).

Since the PLA and PI could also act as a triboelectric (TE) pair and may tend to exchange electrons in the absence of an applied electric field, the off-state behaviour has also been investigated. The result shows that the TE pair could generate a voltage of ∼0.9 V and a charge of ∼0.65 nC, which could be negligible since voltage used to driving the clutch is as high as several hundred volts. The corresponding data are shown in Fig S5.

#### 2.5.1 Effect of thickness

The influence of the dielectric thickness to the shear force of the asymmetric clutch has been investigated. Clutches made from PI with various thickness and PLA with a fixed thickness of 25 μm (PIXPLA25, X refers to the PI thickness, μm) were firstly analyzed. The overlapping area and the applied voltage were set as 4 cm^2^ and 600 V as well. Clutches made from PLA films with variations in thickness and a 5 μm PI film (PLAYPI5, Y represents the thickness of PLA films, μm) have been studied as well. The measured thicknesses of PLA25 and PLA15 films were 23.6 ± 1.1 and 15.0 ± 1.6 μm. The standard deviation of the commercially available PI films was lower than ±0.10 μm. As illustrated in Fig. 2D, the shear force shows a negative correlation with the dielectric thickness and a highest holding force of 18.08 ± 0.78 N (45.21 ± 1.94 kPa) at 600 V could be obtained with the clutch structure of PLA15PI5.

#### 2.5.2 Effect of applied voltage

An asymmetric PLA15PI5 clutch was used to evaluate the V–F relationship. The overlapping area was set at 4 cm^2^. Fig. 2E shows that the clutch responses at 200 V with an electroadhesive force of 1.69 ± 0.07 N and provide a holding force of 24.24 ± 1.01 N (or shear stress of 60.60 ± 2.52 kPa) at 700 V. The fitting result in Fig. 2E displays that the generated shear force increases as V^2.5^ (R^2^ = 0.99), which deviates from the theoretical V^2^ [11] [,^13^] []. The principal reason might be the suppressed air bubbles between the active pads at higher working voltage [12].

#### 2.5.3 Effect of overlapping area

The relationship between the shear force and overlapping area of the PLA15PI5 clutch was also investigated at 600 V. The results are demonstrated in Fig. 2F. The linear fitting result of the curve indicates that the slope is 4.68 N/cm^2^ (or 46.80 kPa) with R^2^ > 0.99, indicating the shear force increases almost linearly with active area, in other word, the shear stress is constant regardless of the variation of the overlapping area.

Fig. 2G demonstrates an electroadhesive clutch on a robotic arm to constrain the rotation and keep the position (both large and small angles) with an overlapping area of 1 × 20 cm^2^ and working voltage of 600 V.

## 3 Conclusions

In this paper, clutches with symmetrical and asymmetrical structures based on PLA and/or PI have been developed and the influence factors responsible for the clutch performance have been studied. The impact of the charge affinity of dielectrics on clutch properties, including electroadhesive force and response time have been evaluated. Provided that the induced charges polarity on the dielectrics are consistent with the charge affinity themselves, the obtained shear force will be significantly increased. Asymmetric clutches with thin dielectric layer, high working voltage and large overlapping area could provide higher shear forces. The PLA15/PI5 clutch could give a shear stress as high as 60 kPa under 700 V with an engage and release time of around 15 and 40 ms. The high shear force of the asymmetric clutch stems from the synergy of electrostatic adhesion and inherent charge characteristics of the dielectrics. Apart from increasing the dielectric constant of the functional layers, this work demonstrates another asymmetric strategy to prepare clutches with high performance.

### Author contribution statement

Jun Li: Conceived and designed the experiments; Contributed reagents, materials, analysis tools or data; Performed the experiments; Analyzed and interpreted the data; Wrote the paper.

Ying Xiong, Bao Yang, Kitming Ma and Linlin Ma: Performed the experiments; Contributed reagents, materials, analysis tools or data.

Xiaoming Tao: Conceived and designed the experiments; Wrote the paper.

### Funding statement

Prof. Xiaoming Tao was supported by Research Grants Council, University Grants Committee [15201419, 15203421], Innovation and Technology Commission - Hong Kong [ITP/041/19TP].

### Data availability statement

Data included in article/supp. material/referenced in article.

### Declaration of interest’s statement

The authors declare no conflict of interest.
