# Supplementary material for: Asymmetric strategy for enhanced performance of flexible electroadhesive clutch
Source: Heliyon. 2023 Jan 12;9(2):e12938. doi: 10.1016/j.heliyon.2023.e12938 (PMC9898634; doi:10.1016/j.heliyon.2023.e12938)
Supplement: Multimedia component 1 [file mmc1.docx]

**Supporting Information**

# Asymmetric Strategy for Enhanced Performance of Flexible Electroadhesive Clutch

Jun Li, Ying Xiong, Kitming Ma, Bao Yang, Linlin Ma and Xiaoming Tao*

Research Institute of Intelligent Wearable Systems

Institute of Textiles and Clothing

The Hong Kong Polytechnic University, Hung Hom, Hong Kong, China.

E-mail: [xiao-ming.tao@polyu.edu.hk](mailto:xiao-ming.tao@polyu.edu.hk)

**Experimental section**

**Materials**

In this paper, PLA and PI were adopted as the functional materials. PLA is easy to retain positive charges while PI tends to hold negative charges. Conductive fabric (3M, 120 μm) with conductive adhesive layer was used as the flexible electrode. The PI films and PLA powder were purchased from Rayitek Co. Ltd, Shenzhen and Nature Works respectively. The thickness of PI ranges from 5 to 50 μm.

**Procedures for the sample preparation**

The PLA powder was firstly recrystalized from hot ethanol to remove impurities. A CHCl_3_ solution of the purified PLA (0.1g/mL) was used for blade coating on an iron plate with a *k* control coater. The blade-coated PLA films were put into oven at 65 ^o^C for 3 hours to fully remove solvents and then peeled off from the substrate. The PLA and PI films were bond to the textile electrodes directly. Then the samples were cut into different shapes accordingly by a rotary cutter.

**Materials characterization**

A ZYGO laser interferometric non-contact profile system was used to explore the roughness of PLA and PI. The sample size was 2 x 2 cm^2^. SEM images were recorded on the JSM-6490 machine. The RK2674A voltage withstand test instrument was used as the DC source. The dielectric constant and loss were measured on a Agilent 4294A precision impedance analyzer.

**Procedures for the dielectric measurement**

The size of the sample was about 1 x 1 cm^2^ and silver paste was used as the electrode for the measurement. The capacitance and dielectric loss between the frequency range of 20 Hz to 1M Hz were measured.

**Procedures for the shear force measurement**

The measurement for the shear force was conduct under high direct-current (DC) voltages. When the two active pads were engaged under an applied voltage, the load cell started to lift quickly at a velocity of 40 N/min. The load at the slip of the two active pads was recorded as the electroadhesive force. After each test, the surfaces of the two pads were neutralized with a static eliminator (SJ-M200) and wiped with ethanol and placed at ambient environment for 5 minutes to eliminate the space charges on the surfaces of two pads.[1] Five groups of data were recorded for each point to give the average and standard deviation values. All tests were conducted under constant experimental condition (21 ^o^C and 40% ~ 50% relative humidity).

**Procedures for the engage and release time**

The experimental setup for the engage and release time is shown in Fig S2. The resistance of R1 and R2 were 0.5 MΩ and 15.0 MΩ. In this setup, the Instron will record the data from both force sensor and voltage sensor at the same time. To measure the engage time, the upper fixture went up slowly (2 mm/min) at first, several seconds later the power was switched on to engage the clutch. For the measurement of release time, the clutch was firstly engaged, then the voltage was turned off. The load cell lifts at a slow velocity (0.001 N/min) to avoid the force drop during the test.

**Procedures for the measurement of triboelectric properties**

The triboelectric behaviour of the PLA/PI pair was conducted on a Keyboard Life Tester (ZX-A03) and the output signals including voltage (V), current (I) and charge (Q) were recorded by Keithley 6514 electrometer. A fixed force of ~ 10 N with a frequency of 3 Hz was applied to generate the triboelectric signals. The overlapping area of the TENG pair was set as 4 cm^2^, which was the same as the size of the clutch used in most measurements.

**Comparison with other work**

The asymmetric clutch could give a shear stress of ~60 kPa with a 15 μm PLA and a 5 μm PI pads under 700 volt, which results from a synergy effect of electroadhesion and charge characteristics. The asymmetric clutch could provide a much higher holding force than those made from dielectric materials with similar dielectric constant^[2]^, and are comparable with those made from high-*k* composites.^[3, 4]^ Though the performance is not as high as the high-*k* polymer based clutch, it is expected to further increase the shear force if we could have high-*k* polymers with opposite charge affinities.

## References:

[1] T. Nakamura, A. Yamamoto, *ROBOMECH J.* **2017**, *4*, 18.

[2] R. Hinchet, V. Vechev, H. Shea, O. Hilliges, in *31st Annu. ACM Symp. User Interface Softw. Technol. - UIST ’18*, ACM Press, New York, New York, USA, **2018**, 901.

[3] V. Ramachandran, J. Shintake, D. Floreano, *Adv. Mater. Technol.* **2019**, *4*, 1800313.

[4] S. B. Diller, S. H. Collins, C. Majidi, *J. Intell. Mater. Syst. Struct.* **2018**, *29*, 3804.


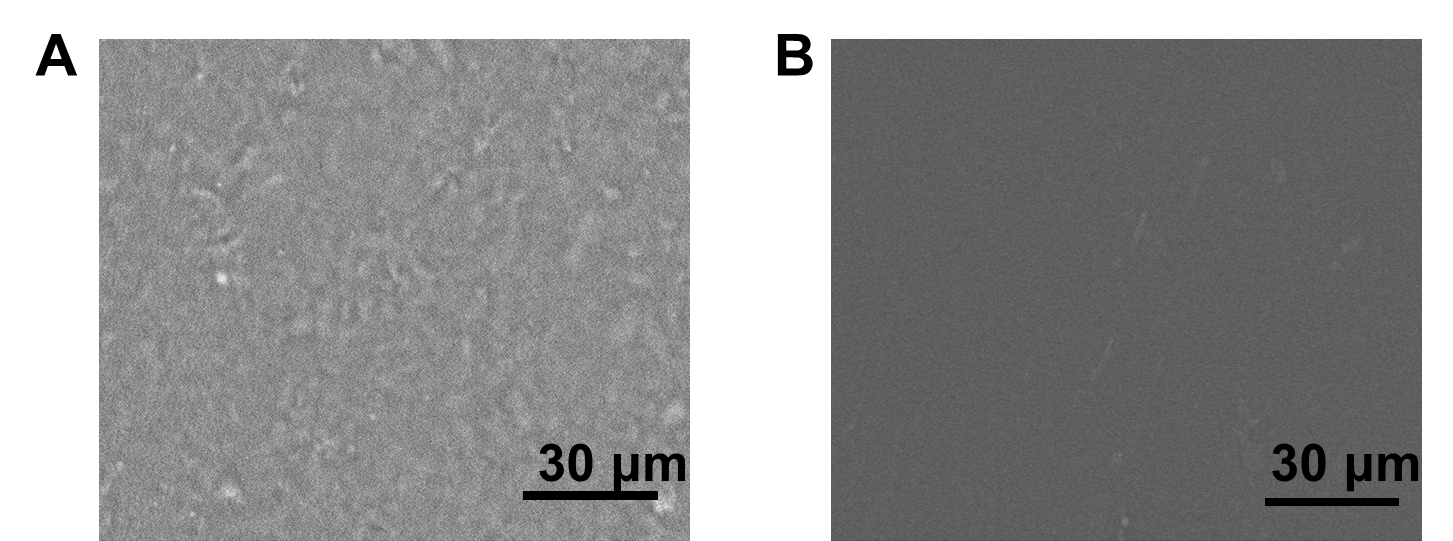


**Figure S1**. SEM images of (A) PLA and (B) PI film


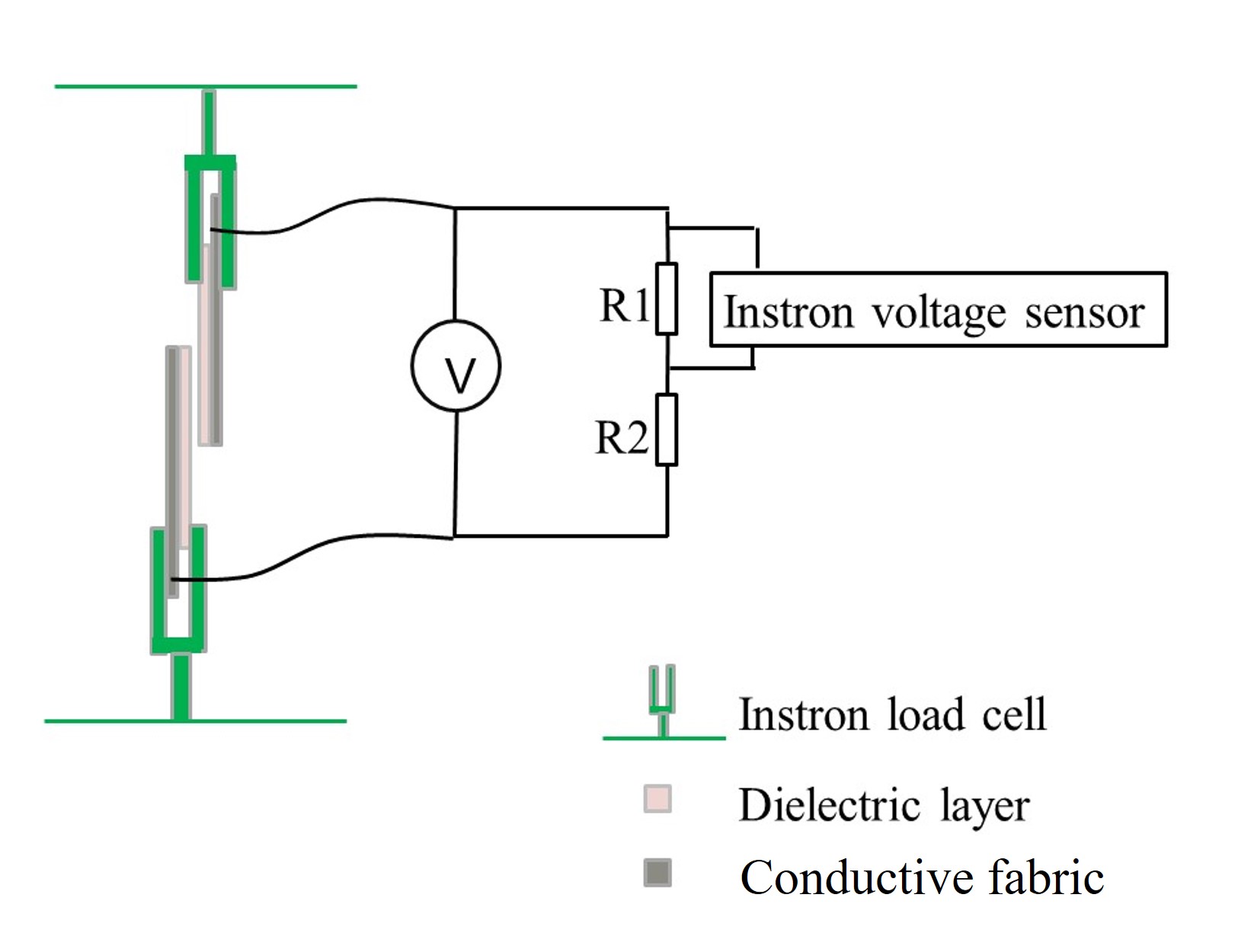


**Figure S2.** Schematic illustration of the test system


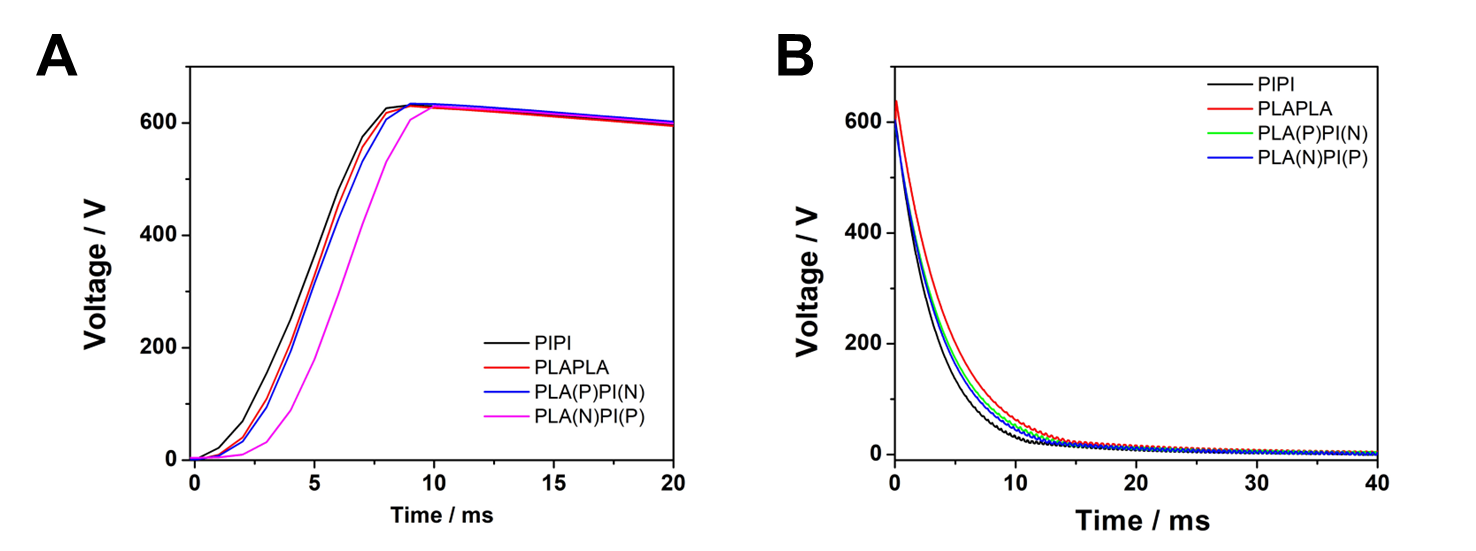


**Figure S3.** Indirect method of (A) engage and (B) release time.


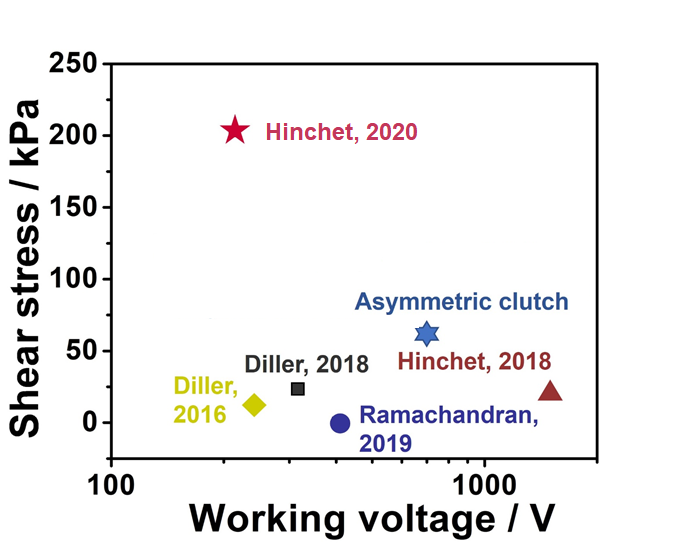


**Figure S4.** Benchmarks with other electroadhesive clutches


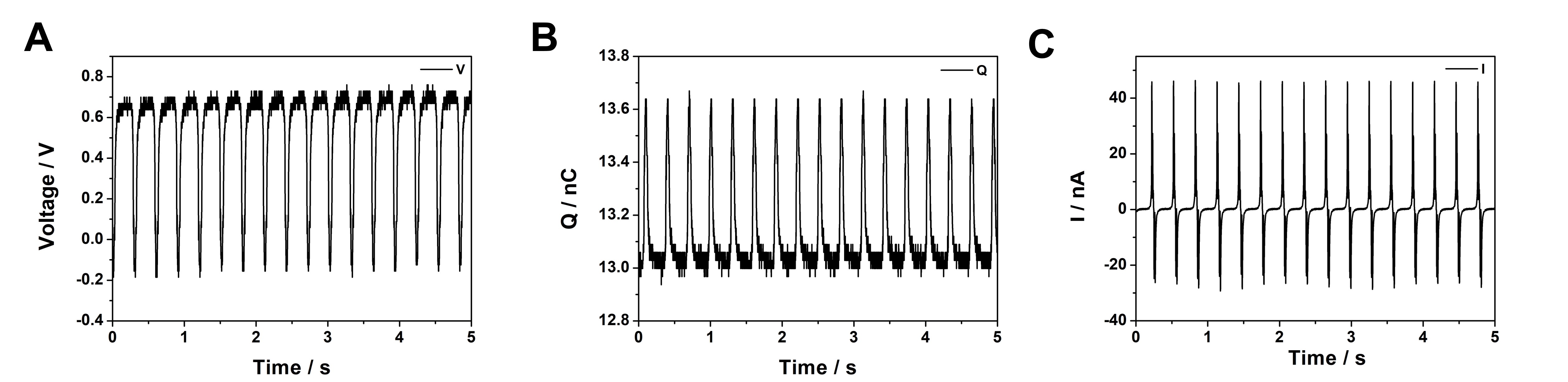


**Figure S5.** Triboelectric properties of the PLA/PI pair (A = 4 cm^2^, F = 10 N, f = 3 Hz)


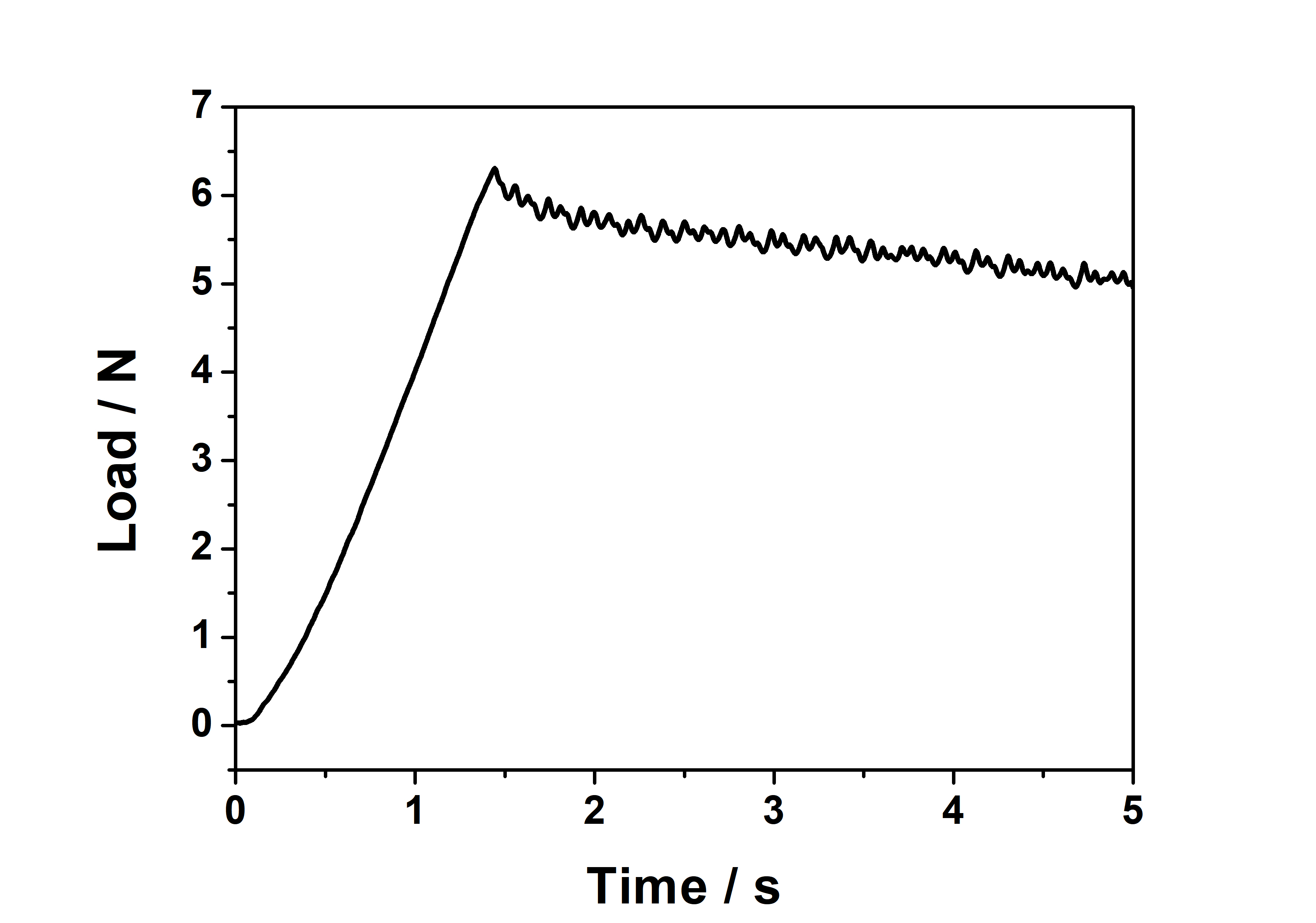


**Figure S6.** Measured load of PLA(N)PI(P) clutch at 600 V
